# ω-Methylsulfanylalkyl Glucosinolates: A General Synthetic Pathway

**DOI:** 10.3390/molecules23040786

**Published:** 2018-03-28

**Authors:** Manolis Mavratzotis, Stéphanie Cassel, Sabine Montaut, Patrick Rollin

**Affiliations:** 1 Institut de Chimie Organique et Analytique (ICOA), Université d’Orléans et CNRS, UMR 7311, BP 6759, F-45067 Orléans CEDEX 2, France; mavratzotis.manolis@gr.sika.com (M.M.); cassel@chimie.ups-tlse.fr (S.C.); 2 Laboratoire IMRCP, UMR CNRS 5623, Université P. Sabatier Toulouse III, 118 route de Narbonne, 31062 Toulouse CEDEX 9, France; 3 Department of Chemistry and Biochemistry, Biomolecular Sciences Programme, Laurentian University, Sudbury, ON P3E 2C6, Canada; smontaut@laurentian.ca

**Keywords:** glucosinolate, thiohydroximate, thiofunctionalized, nitronate

## Abstract

A general pathway was devised to synthesize ω-methylsulfanylalkyl glucosinolates, which represent an important class of structurally homogeneous plant secondary metabolites. The required thiofunctionalized hydroximoyl chlorides were obtained from the corresponding α,ω-nitroalkyl methylsulfide precursors, involving as the key-step, a nitronate chlorination strategy. A coupling reaction with 1-thio-*beta*-d-glucopyranose, followed by *O*-sulfation of the intermediate thiohydroximate and final deprotection of the sugar moiety afforded the target compounds.

## 1. Introduction

**Figure 1:**
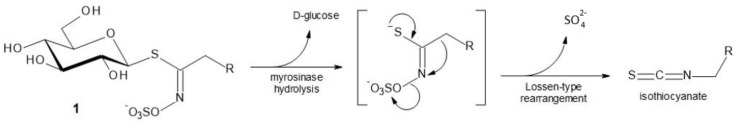
Glucosinolates (GLs) and their enzymatic degradation.

Glucosinolates (GLs) **1** stand as molecular tags of the plant order Brassicales [1,2]. Those strikingly bio-relevant thiosaccharidic secondary metabolites are found without exception in all 17 families of this order. All of the known GLs (ca. 130 molecules to date) display a remarkable structural homogeneity based on a hydrophilic *beta*-d-glucopyrano unit, which bears a *O*-sulfated anomeric (*Z*)-thiohydroximate function connected to a rather hydrophobic side chain R. Depending on plant species, the constitution of R is the sole structural variant, in which diversified aliphatic, arylaliphatic, or indolic arrangements can be found. Associated in plants with an atypical glycohydrolase, myrosinase (E.C. 3.2.1.147), GLs can undergo hydrolytic cleavage of the anomeric C–S bond. After d-glucose release, the detached aglycon undergoes a Lossen-type rearrangement to produce biologically active isothiocyanates (Figure 1) [1,2,3]. GLs can thus be regarded as isothiocyanate bio-precursors.

**Figure 2:**
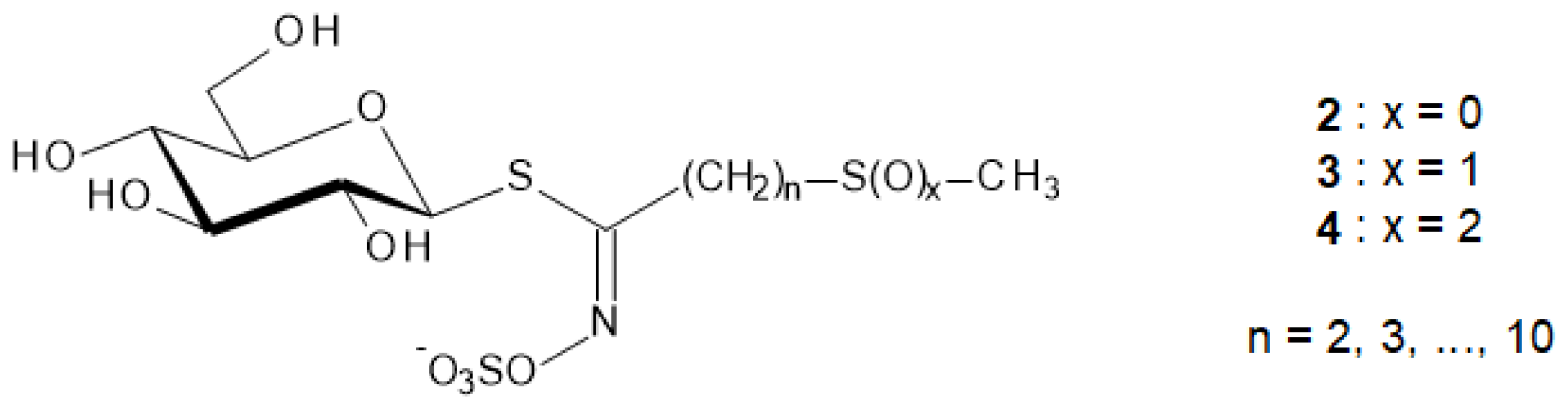
Glucosinolates bearing an ω-thiofunctionalized side chain [4,5].

It is remarkable that more than one-third of the actually registered GL structures display an additional thio-function—namely sulfide (**2**), sulfoxide (**3**) or sulfone (**4**)—in ω-position of their aglycon chain, as represented in Figure 2 [4,5].

Mainly found in the *Brassicaceae* and *Capparaceae* families, such thiofunctionalized GLs have been investigated over the last decade for their redox properties in various in vitro free radical-scavenging assays, with a view to assessing their antioxidant potential [6,7,8].

Notwithstanding the large number of known GL-containing plant species, accessing these compounds from appropriate vegetable sources via extractive processes is generally not straightforward [9,10]. “Specialist” plants, which can be considered for convenient isolation of one specific GL in reasonable amount, are in very limited number [11,12,13]. This is particularly the case for thiofunctionalized GLs, for which the synthetic approach should be privileged to make available attractive substrates for biological studies [14,15]. Nevertheless, since our pioneering work [16], only a small number of syntheses have focused on GLs bearing an external thio-function in the aglycon part. Starting from a sulfoxide-oxime precursor, isotopically labelled 4-methylsulfinylbutyl GL (glucoraphanin) was synthesized by Botting et al. for analysis and metabolic studies [17,18]. 4-Methylsulfanylbut-3-enyl GL (glucoraphasatin) and ^13^C-labelled 4-methylsulfanylbutyl GL (glucoerucin) were also synthesized [19,20].

**Figure 3:**
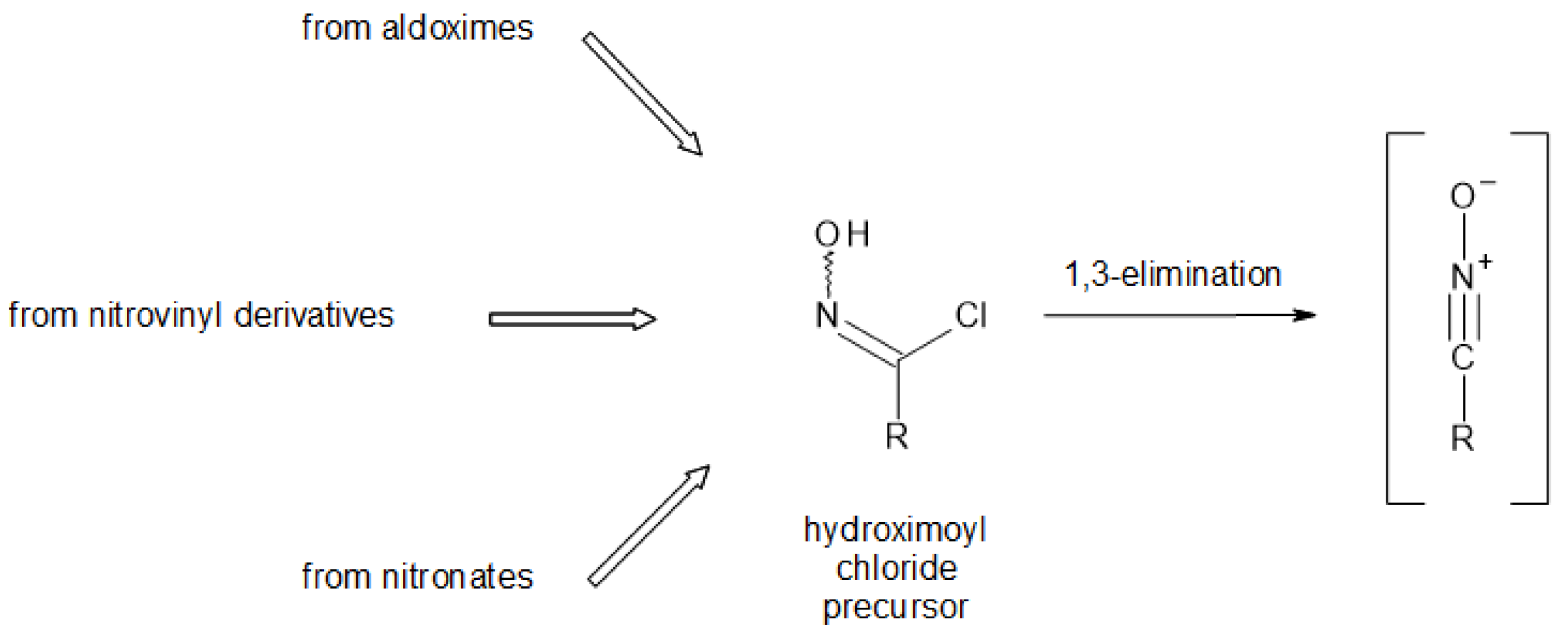
Methods to generate nitrile oxides [15].

For most synthetic methods that have been developed for GLs over the years, the key step was the stereospecific formation of a thiohydroximate function, usually resulting from the 1,3-addition of a glucosyl mercaptan onto a transient nitrile oxide [15]. As they are generally unstable molecular species, nitrile oxides have to be generated in situ through base-induced conversion of hydroximoyl chlorides, (Figure 3), which in turn currently result from electrophilic chlorination of aldoximes [21].

In this particular case, however, the presence on the carbon chain of a methylsulfanyl moiety *a priori* precludes the use of reagents like chlorine or *N*-chlorosuccinimide which are prone to oxidize a sulfide function [22].

**Scheme 1:**
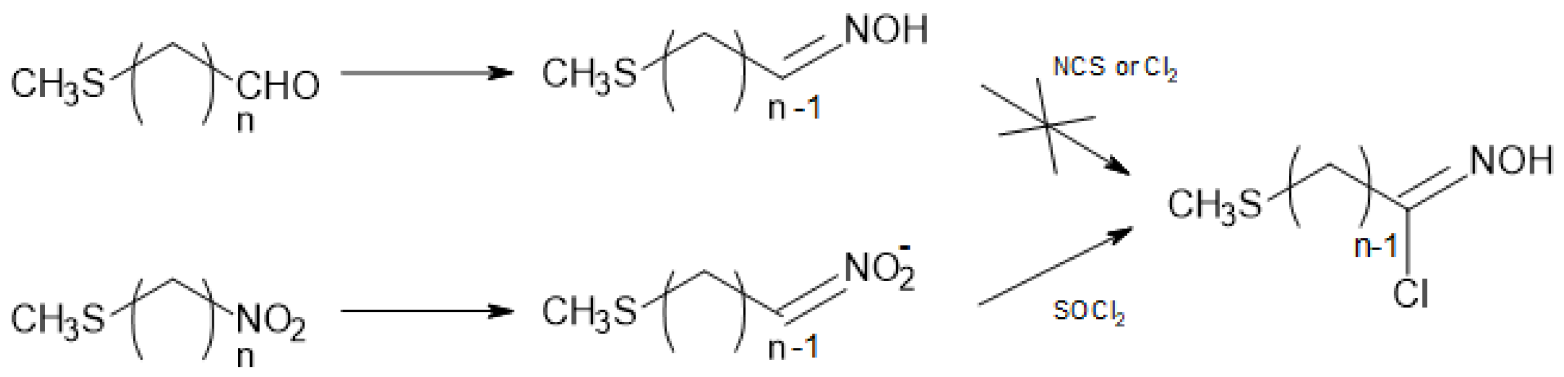
Nitronate method vs. oxime method to access hydroximoyl chlorides [20].

Therefore the alternative nitronate chlorination methodology (Scheme 1) was selected to access the nitrile oxide precursors [15]. In this paper, we report a general synthetic pathway to ω-methylsulfanylalkyl GLs **2** starting from readily prepared ω-nitroalkyl methyl sulfides **5**.

## 2. Results and Discussion

The preparation of a range of linear ω-nitroalkyl methyl sulfides **5** of variable length (n = 3–12) from commercially available ω-bromochloroalkanes **6** was expected to be straightforward. However, in the case of small sized polymethylene chain (n = 3–6), undesirable side-reactions took place. Hence, distinct strategies had to be devised to provide the ω-nitroalkyl methyl sulfide precursors **5**.

**Scheme 2:**
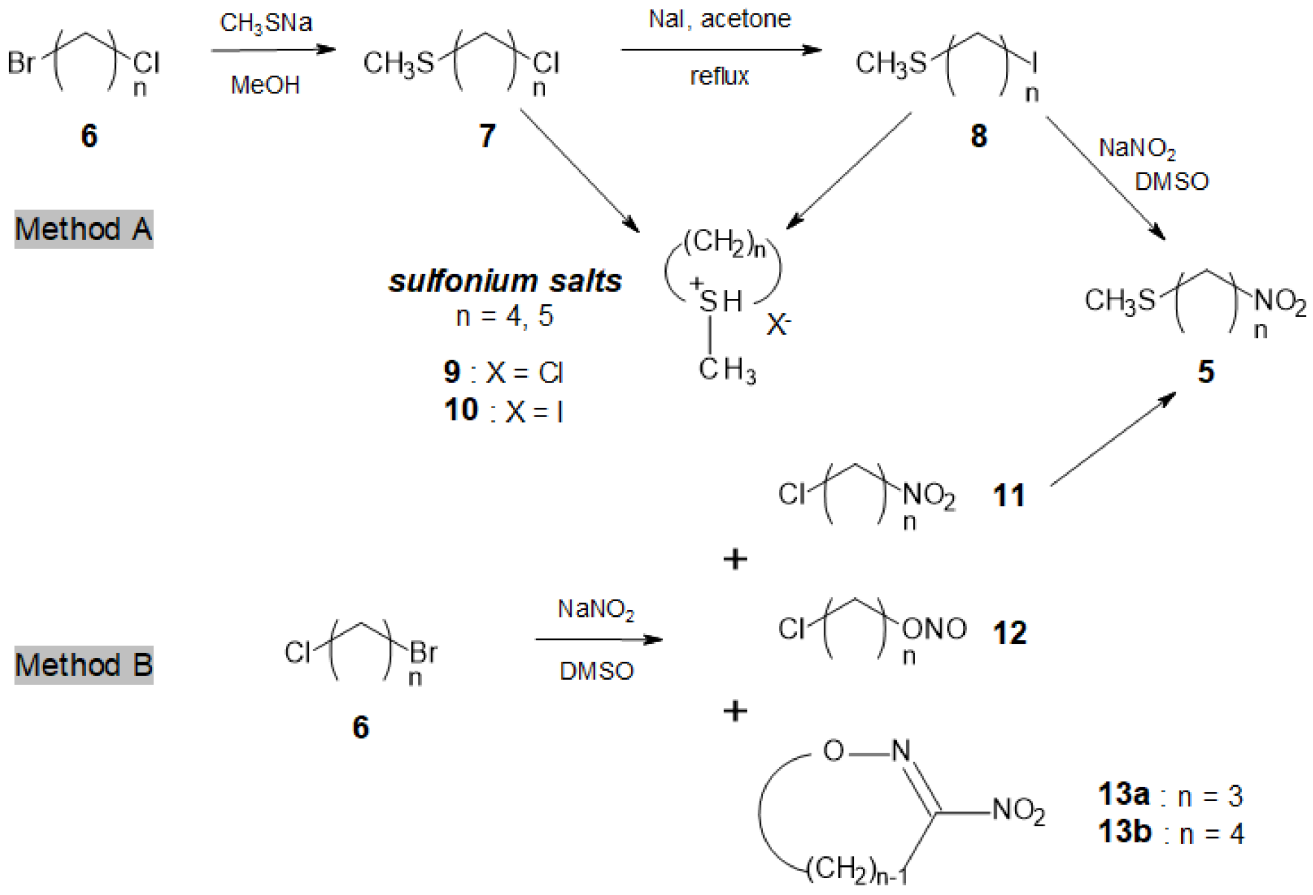
Access to small-sized ω-nitroalkyl methyl sulfides **5a**–**d**.

Method A (Scheme 2) is suggested for dihaloalkanes **6a** (n = 3) or **6d** (n = 6). In the cases of **6b** and **6c** (n = 4 and 5), the reaction sequence was almost disrupted because of the formation of cyclic sulfonium salts **9** and **10** [23,24].

Compounds **7** and **8** have a tendency to undergo cyclization during their formation, on standing or/and during their purification by distillation. Sulfonium salts formation rate is carbon chain length-, temperature-, and halogen type-dependent. Thus, rapid cyclization occurred in the case of ω-methylsulfanyl-iodobutane **8b** and -pentane **8c**. Slightly slower, but still significant, cyclization took place with the corresponding chloro-analogues, ω-methylsulfanyl-chlorobutane **7b** and -pentane **7c** while in the case of 6-methylsulfanyl-1-chlorohexane **7d**, the cyclization was minimized (Table 1). Halogen-exchange reaction of compounds **7** with iodide ion was very slow, even at an elevated temperature, thus explaining the result reported in Table 1 for n = 4 or 5. In cases where n = 3 or 6, chloro- and iodo-compounds were more stable—enough at least for completing the method A reaction sequence and afford the desired α,ω-nitro-sulfides **5**.

α,ω-*Bis*-methylsulfanylalkanes can be simultaneously formed [25] during the preparation of compounds **7**. Their occurrence can be minimized by controlling the temperature, the reaction time, and the molar ratio of CH_3_SNa. Nevertheless, even if they are not separated from the reactive intermediates, the presence of such side-products is not likely to disturb the subsequent reaction steps.

According to method B (Scheme 2), reaction of NaNO_2_ with ω-bromo-chloroalkanes **6** afforded the ω-chloro-nitroalkanes **11** together with the isomeric nitrites **12** [27,28]. The reaction needed to be time-controlled for each dihaloalkane **6** in order to optimize the nitro-compound to nitrite ratio (Table 2), and at the same time to prevent destruction of the nitro-derivatives **11** due to the simultaneous presence of the nitrite ion and an alkyl nitrite [29].

In the case of **6a** (n = 3), notably, the formation in ca. 40% yield of the known 3-nitro-2-isoxazoline **13a** occurred, because of the interaction of the nitro-derivative **11a** with the nitrite **12a** [30]. In fact, a prolonged reaction of 1,3-bromochloropropane with NaNO_2_ in the presence of 1-propyl nitrite has been patented [31] as a preparative method for **13a** [32]. In contrast in the case of **5b** (n = 4), no side-formation of the corresponding oxazine **13b** was observed.

The ω-chloro-nitroalkanes **11a**–**d** were isolated by flash chromatography and were characterized by GC-MS and NMR analysis. Subsequent conversion of **11a**–**d** into the expected ω-methylsulfanyl-nitroalkanes **5a**–**d** was carried out by reaction with freshly prepared sodium methanethiolate, obtained by passing CH_3_SH gas in a 1 M methanolic solution of sodium methoxide. Indeed, commercial powdered CH_3_SNa inevitably gave poor conversion yields and produced a number of unexpected side products (acids, esters, lactones, thiosulfonates...), e.g., a high yield of γ-butyrolactone in the case of **11b** (n = 4).

After their formation, ω-methylsulfanyl-nitroalkanes **5** had to be recovered from the basic reaction medium by the addition of a protic acid. However the regeneration of aliphatic nitro-compounds from their salts is not a routine matter [33]. Depending on the experimental conditions, acidic treatment of primary nitronates often results in the production of aldehydes and/or hydroxamic acids via Nef and Meyer reactions [34]. Optimization of nitroalkane recovery is dependent on resorting to mild acidification protocols, such as hydroxylamine hydrochloride in 10% aqueous acetic acid at low temperature. Applying such conditions in the knotty case of **11b** (n = 4) allowed to satisfactorily recover (1-methylsulfanyl)-4-nitrobutane **5b**, without the formation of γ-butyrolactone.

**Scheme 3:**
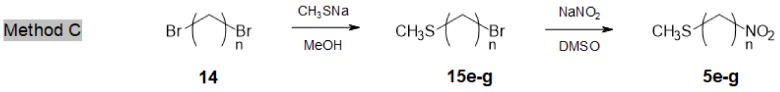
Access to longer chain ω-methylsulfanyl nitroalkanes **5e**–**g**.

Access to longer chain ω-methylsulfanyl-nitroalkanes **5e**-**g** (n = 8, 10, 12) was more straightforward (Method C, Scheme 3). Commercially available α,ω-dibromoalkanes **14** were reacted with CH_3_SNa to afford ω-methylsulfanyl bromoalkanes **15e**-**g** (#60% yield), together with minor amounts of the corresponding α,ω-bis-methylsulfanylalkanes [25]. Finally, compounds **15e**-**g** were converted, as described above, into the desired ω-methylsulfanyl nitroalkanes **5e**-**g** in 65–70% yields.

**Scheme 4:**
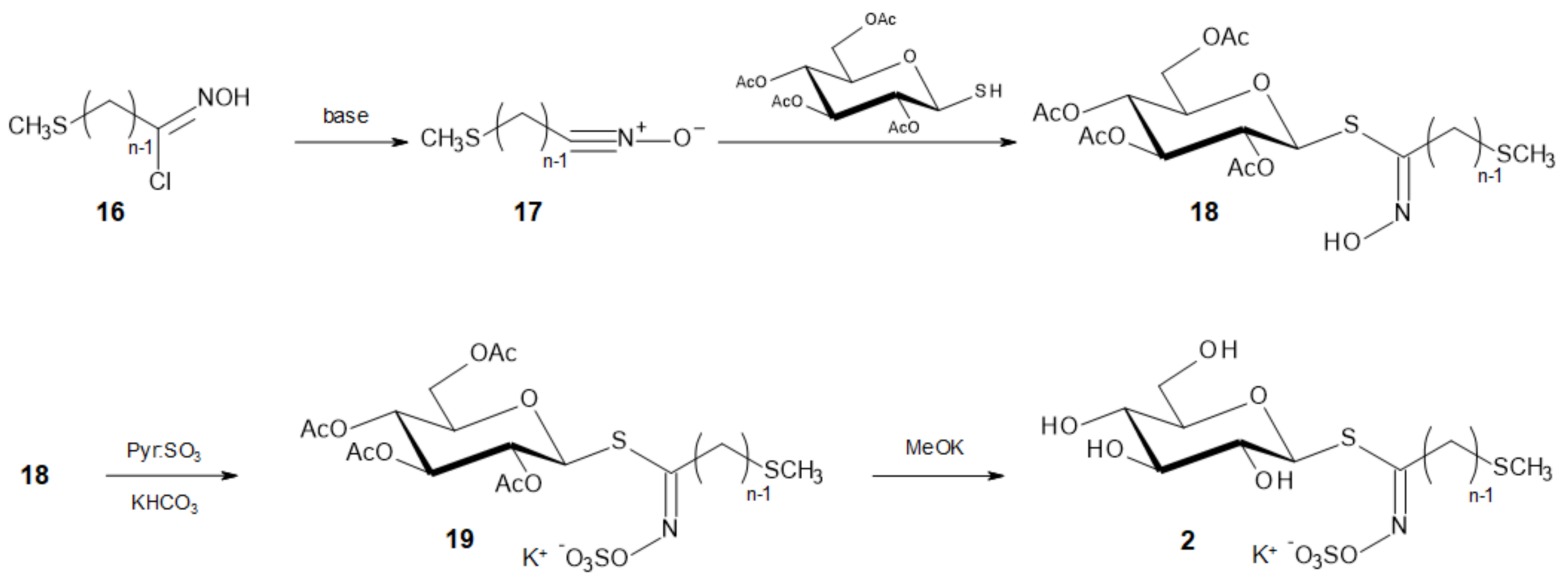
Coupling reaction to deliver tetra-*O*-acetylated ω-methylthioalkyl (*Z*)-thiohydroximates **18**, followed by *O*-sulfation and deprotection to yield GLs **2**.

Following a procedure previously established by Kjaer [35,36], all of the nitro-compounds **5** were converted into their moisture-sensitive sodium salts, which were isolated by brief filtration under dry atmosphere, then reacted in chloroform with SOCl_2_ (in our hands, the oxalyl chloride variant [37] did not bring substantial improvement) at low temperature (−60 °C) to afford the hydroximoyl chlorides **16**. Those were subsequently used without further purification in the next steps of the synthesis (Scheme 4).

The intermediate nitrile oxides **17** were generated in situ by base-catalyzed (NEt_3_) 1,3-elimination on hydroximoyl chlorides **16** to react [15,38] with 2,3,4,6-tetra-*O*-acetyl-1-thio-β-d-glucopyranose [39] to afford the corresponding ω-methylthioalkyl (*Z*)-thiohydroximates **18**.

Final conversion of the thiohydroximates **18a**–**g** into the target GLs **2a**–**g** was performed according to a previously described protocol [15]. *O*-Sulfation of the hydroximino moiety using sulfur trioxide pyridine complex, followed by quenching with aqueous potassium hydrogenocarbonate afforded the per-*O*-acetylated GLs **19a**–**g**, the carbohydrate part of which was readily deprotected under basic conditions to afford the corresponding GLs.

## 3. Experimental Section

### 3.1. General Information

α,ω-Bromochloroalkanes **6a**–**d** Br-(CH_2_)_n_-Cl (n = 3–6) and α,ω-dibromoalkanes **14** Br-(CH_2_)_n_-Br (n = 8, 10, 12) were obtained from commercial sources (Sigma-Aldrich, Saint-Quentin-Fallavier, France) and were used without further purification. Anhydrous reactions were performed under argon atmosphere in pre-dried flasks, using distilled anhydrous solvents. Unless otherwise stated, all of the chemicals obtained from Sigma-Aldrich were used without further purification. TLC on precoated aluminum-back plates (Merck Kieselgel 60F254, E. Merck, Darmstadt, Germany) were generally visualized by UV light (254 nm) and by charring after exposure to a 5% H_2_SO_4_ solution in ethanol. Flash column chromatography was carried out using silica gel (E. Merck, 36–63 mesh, unless otherwise indicated). Melting points (°C) were obtained using a Büchi 510 apparatus (Büchi Sarl, Rungis, France) and are uncorrected. Optical rotations were measured at 20 °C with a Perkin Elmer 341 polarimeter (Perkin Elmer France, Villebon s/Yvette, France) with a path length of 1 dm. ^1^H and ^13^C-NMR spectra were recorded on 250 MHz Bruker Avance DPX250 or 400 MHz Bruker Avance2 spectrometers (Bruker France, Wissembourg, France). Chemical shifts are expressed in parts per million (ppm) downfield from TMS internal standard and coupling constants are given in Hz. NMR peak assignments were established using NOESY, COSY, HSQC, and HMBC methods for all the reported compounds.

GC monitoring was carried out using a Varian 3700 gas chromatograph (Agilent Technologies, Les Ulis, France) equipped with a split/splitess injector at 240 °C, a DB-5 (Agilent) capillary column (30 m × 0.25 mm, 0.25 µm film thickness—carrier gas nitrogen 1 mL·min^−1^) and a flame ionization detector at 280 °C. Depending on the solute analyzed, two temperature programs were used: (1) initial temperature 100 °C, held for 2 min, and then ramped to 220 °C with a 10 °C·min^−1^ rate maintained for 10 min. (2) initial temperature 100 °C, held for 2 min, then ramped to 250 °C with a 15 °C·min^−1^ rate maintained for 15 min.

GC-MS analyses were performed using a HP-5890 (Agilent) chromatograph hyphenated to a HP-5970 mass spectrometer (Agilent quadrupole). Spectra were obtained in EI mode at 70 eV. The gas chromatograph was equipped with a HP-1 capillary column (Agilent, 100% dimethylsiloxane, 30 m × 0. 25 mm, 0.33 µm film thickness). The injector temperature was held at 200 °C and a 1 µL sample was injected with a 1/100 split ratio under a constant helium flow (1 mL·min^−1^). The oven temperature was initially held at 60 °C for 3 min, and then ramped with a 3 °C·min^−1^ rate to 80 °C. This temperature was held for 1 min, then ramped with a 3 °C·min^−1^ rate to 150 °C. A 5 °C·min^−1^ rate was used to bring the oven temperature to 200 °C. The final temperature of 230 °C was reached with a 10 °C·min^−1^ rate and maintained for 20 min.

Mass spectra (ion spray; IS) were recorded on a Perkin–Elmer Sciex API-300 spectrometer (Sciex Sarl, Les Ulis, France).

High resolution mass spectra (HRMS) were recorded with a MicrOTOF-QII spectrometer (Bruker France S.A.S., Champs-sur-Marne, France) in the electrospray ionisation (ESI) mode.

### 3.2. Synthesis

#### 3.2.1. General Procedure for the Synthesis of Small-Sized Chloroalkyl Methyl Sulfides **7a**–**d**

A 2.6 M methanolic solution of MeSNa (23 mL, 1.2 equiv.) was added dropwise to a stirred solution of ω-bromochloroalkane **6** (50 mmol) in dry methanol (80 mL) at room temperature (r.t.). Stirring was continued until complete consumption of the dihalide (2–3 h, GC monitoring). The mixture was concentrated in vacuo and the resulting white slurry was taken in cold water and extracted with dichloromethane. After drying the organic phase over MgSO_4_, filtration and concentration in vacuo, the oily residue was purified by flash chromatography using silica gel (230–400 mesh) with hexane as eluent to afford **7a**–**d** as light yellow oils.

ω-Chloro-1-methylsulfanylpropane **7a** [24,40], -butane **7b** [24], -pentane **7c** [24].

6-Chloro-1-methylsulfanylhexane [98429-85-7] **7d** [41,42].

#### 3.2.2. General Procedure for the Synthesis of Long-Sized Bromoalkyl Methylsulfides **15e**–**g**

Similarly, the α,ω-dibromoalkane **14** (50 mmol) dissolved in a stirred methanol (200 mL)-dichloromethane (50 mL) mixture was treated dropwise (over 30 min) by a 2.6 M methanolic solution of MeSNa (20 mL, 1.04 equiv.), while cooling at r.t. After complete consumption of the dihalide (1–2 h, TLC monitoring), the mixture was concentrated in vacuo and the resulting white slurry was taken in cold water and extracted with dichloromethane. After drying the organic phase over MgSO_4_, filtration and concentration in vacuo, the oily residue was purified by flash chromatography using silica gel (230–400 mesh) with hexane as eluent to afford **15e**–**g** [25].

*8-Bromo-1-methylsulfanyloctane [64053-04-9]* **15e** [43]. Light yellow viscous oil (57% yield). ^1^H-NMR (CDCl_3_) δ 1.25–1.44 (m, 8H, CH_2_), 1.57 (qt, 2H, H-2), 1.84 (qt, 2H, H-7), 2.08 (s, 3H, MeS), 2.48 (t, 2H, *J*_vic_ = 7.3, H-1), 3.39 (t, 2H, *J*_vic_ = 6.8, H-8). ^13^C-NMR δ 15.3 (MeS), 27.9, 28.5, 28.9 (C-2-C-6), 32.6 (C-7), 33.8 (C-8), 34.1 (C-1). ESI-MS *m*/*z* 240.2 [M + H]^+^.

*10-Bromo-1-methylsulfanyldecane* **15f**. Light yellow solid (55% yield). ^1^H-NMR (CDCl_3_) δ 1.22–1.47 (m, 12H, CH_2_), 1.58 (qt, 2H, H-2), 1.85 (qt, 2H, H-9), 2.08 (s, 3H, MeS), 2.47 (t, 2H, *J*_vic_ = 7.3, H-1), 3.39 (t, 2H, *J*_vic_ = 6.8, H-10). ^13^C-NMR δ 15.3 (MeS), 28.0, 28.6, 29.0, 29.2 (C-2-C-8), 32.7 (C-9), 33.8 (C-10), 34.1 (C-1). ESI-MS *m*/*z* 268.3 [M + H]^+^.

*12-Bromo-1-methylsulfanyldodecane* **15g**. Light yellow solid (59% yield). ^1^H-NMR (CDCl_3_) δ 1.19–1.48 (m, 16H, CH_2_), 1.58 (qt, 2H, H-2), 1.84 (qt, 2H, H-11), 2.08 (s, 3H, MeS), 2.48 (t, 2H, *J*_vic_ = 7.3, H-1), 3.39 (t, 2H, *J*_vic_ = 6.8, H-12). ^13^C-NMR δ 15.4 (MeS), 28.0, 28.6, 28.7, 29.0, 29.1, 29.3, 29.4 (C-2-C-10), 32.7 (C-11), 33.9 (C-12), 34.2 (C-1). ESI MS *m*/*z* 296.3 [M + H]^+^.

#### 3.2.3. Procedures for the Synthesis of Nitroalkyl Methylsulfides **5**

##### Method A. Synthesis of **5a** (n = 3) and **5d** (n = 6)

The chloroalkyl methyl sulfide **7** (42 mmol) was refluxed in dry acetone with dry NaI (13.5 g, 90 mmol) for 2–3 days until the complete conversion of the starting material (GC monitoring). After concentration in vacuo, the resulting slurry was taken in a 0.6 M aqueous sodium thiosulfate solution, and extracted with CH_2_Cl_2_. The combined organic phases were dried over MgSO_4_, filtered and concentrated in vacuo to afford compound **8** as fragrant pale orange oil, which was used as such in the next step.

To a solution of dry sodium nitrite (2.0 g, 29 mmol) in DMSO (80 mL), a solution of **8** (24 mmol) in DMSO (15 mL) was added dropwise, while keeping the temperature at 20–25 °C. After 2–3 h, the reaction was quenched by adding ice-water (300 mL) and repeatedly extracted with diethyl ether. The combined organic phases were dried over MgSO_4_, filtered and concentrated in vacuo. The residue was purified by silica gel column chromatography (petroleum ether–Et_2_O 4:1) to afford **5a** and **5d** as colourless oils.

*(1-Methylsulfanyl)-3-nitropropane [182258-93-1]* **5a**. Colourless oil (2.27 g, 40% overall yield). ^1^H-NMR (CDCl_3_) δ 2.09 (s, 3H, MeS), 2.27 (qt, 2H, H-2), 2.58 (t, 2H, *J*_vic_ = 6.8, H-1), 4.51 (t, 2H, *J*_vic_ = 6.8, H-3). ^13^C-NMR δ 15.1 (MeS), 26.1 (C-2), 30.4 (C-1), 73.7 (C-3). MS IS *m*/*z* 136.2 [M + H]^+^.

*(1-Methylsulfanyl)-6-nitrohexane* **5d**. Colourless oil (3.13 g, 42% overall yield). ^1^H-NMR (CDCl_3_) δ 1.32–1.48 (m, 4H, H-3, H-4), 1.54–1.63 (m, 2H, H-2), 1.94–2.04, m, 2H, H-5), 2.06 (s, 3H, MeS), 2.46 (t, 2H, *J*_vic_ = 7.3, H-1), 4.36 (t, 2H, *J*_vic_ = 7.3, H-6). ^13^C-NMR δ 15.2 (MeS), 25.6 (C-4), 27.0 (C-5), 27.7 (C-3), 28.4 (C-2), 33.8 (C-1), 75.5 (C-6). MS IS *m*/*z* 178.3 [M + H]^+^.

##### Method B. Synthesis of **5a**–**d** (n = 3–6) from ω-bromochloroalkanes **6a**–**d**

To a solution of dry sodium nitrite (1.24 g, 18 mmol) in DMSO (100 mL), a solution of ω-bromochloroalkane **6** (15 mmol) in DMSO (20 mL) was added dropwise while keeping the temperature at 20–25 °C. After 2–5 h, the reaction was quenched by adding ice-water (400 mL) and was repeatedly extracted with diethyl ether. The combined organic phases were dried over MgSO_4_, filtered and concentrated in vacuo. The residue was purified by column chromatography (petroleum ether–Et_2_O 5:1) to afford ω-chloronitroalkanes **11** as colourless oils.

*1-Chloro-3-nitropropane [16694-52-3]* **11a**. Colourless oil (0.8 g, 42% yield) ^1^H-NMR (CDCl_3_) δ 2.45 (qt, 2H, H-2), 3.65 (t, 2H, *J*_vic_ = 6.3, H-1), 4.58 (t, 2H, *J*_vic_ = 6.3, H-3). ^13^C-NMR δ 29.6 (C-2), 40.7 (C-1), 72.1 (C-3) [44].

*1-Chloro-4-nitrobutane [41168-66-5]* **11b**. Colourless oil (1.2 g, 60% yield) ^1^H-NMR (CDCl_3_) δ 1.83–1.92 (m, 2H, H-2), 2.13–2.23 (m, 2H, H-3), 3.58 (t, 2H, *J*_vic_ = 6.1, H-1), 4.43 (t, 2H, *J*_vic_ = 6.8, H-4). ^13^C-NMR δ 24.4 (C-2), 28.8 (C-3), 43.5 (C-1), 74.6 (C-4) [45].

*1-Chloro-5-nitropentane [1173694-33-1]* **11c**. Colourless oil (1.5 g, 66% yield) ^1^H-NMR (CDCl_3_) δ 1.51–1.60 (m, 2H, H-3), 1.77–1.87 (m, 2H, H-2), 1.98–2.08 (m, 2H, H-4), 3.54 (t, 2H, *J*_vic_ = 6.3, H-1), 4.39 (t, 2H, *J*_vic_ = 7.0, H-5). ^13^C-NMR δ 23.4 (C-3), 26.4 (C-4), 31.5 (C-2), 44.2 (C-1), 75.3 (C-5).

*1-Chloro-6-nitrohexane [898543-32-3]* **11d**. Colourless oil (1.9 g, 76% yield) ^1^H-NMR (CDCl_3_) δ 1.38–1.55 (m, 4H, H-3, H-4), 1.73–1.83 (m, 2H, H-2), 1.97–2.07 (m, 2H, H-5), 3.53 (t, 2H, *J*_vic_ = 6.6, H-1), 4.38 (t, 2H, *J*_vic_ = 7.0, H-6). ^13^C-NMR δ 25.4 (C-4), 25.9 (C-3), 27.0 (C-5), 31.9 (C-2), 44.6 (C-1), 75.4 (C-6) [46].

A solution of chloronitro derivative **11** (25 mmol) in dry methanol (50 mL) was reacted with a 2.6 M methanolic solution of MeSNa (20 mL, 2.08 equiv.) under the conditions that are reported in Table 3. After concentration in vacuo, the resulting slurry was poured into ice water (200 mL) under vigorous stirring and treated dropwise at 0 °C by a cooled solution of hydroxylamine hydrochloride (4.0 g, 57 mmol) in 20% aqueous acetic acid (17 mL) [28]. The two-phase system obtained was extracted three times with CH_2_Cl_2_, and the combined organic phases were dried over MgSO_4_, filtered and concentrated in vacuo. The residue was purified by column chromatography (petroleum ether–Et_2_O 4:1) to afford **5a**–**d** as colourless oils (**5a** and **5d** were described above).

*(1-Methylsulfanyl)-4-nitrobutane [182258-94-2]* **5b**. Colourless oil (1.6 g, 43% yield). ^1^H-NMR (CDCl_3_) δ 1.63–1.73 (m, 2H, H-2), 2.03–2.16 (m, 5H, MeS, H-3), 2.52 (t, 2H, *J*_vic_ = 7.0, H-1), 4.40 (t, 2H, *J*_vic_ = 7.0, H-4). ^13^C-NMR δ 15.1 (MeS), 25.2 (C-2), 25.9 (C-3), 33.0 (C-1), 75.0 (C-4). MS IS *m*/*z* 150.2 [M + H]^+^.

*(1-Methylsulfanyl)-5-nitropentane [182258-95-3]* **5c**. Colourless oil (3.5 g, 86% yield). ^1^H-NMR (CDCl_3_) d 1.42–1.52 (m, 2H, H-3), 1.58–1.68 (m, 2H, H-2), 1.96–2.06 (m, 2H, H-4), 2.07 (s, 3H, MeS), 2.48 (t, 2H, *J*_vic_ = 7.1, H-1), 4.37 (t, 2H, *J*_vic_ = 7.1, H-5). ^13^C-NMR δ 15.2 (MeS), 25.0 (C-3), 26.7 (C-4), 28.00 (C-2), 33.5 (C-1), 75.3 (C-5). MS IS *m*/*z* 164.2 [M + H]^+^.

##### Method C. Synthesis of Long-Sized Nitroalkyl Methylsulfides **5e**–**g** (n = 8, 10, 12)

To a solution of dry sodium nitrite (2.0 g, 29 mmol) in DMSO (80 mL), a solution of **15** (24 mmol) in DMSO (15 mL) was added dropwise, while keeping the temperature at 20–25 °C. After 2–3 h, the reaction was quenched by adding ice-water (300 mL) and repeatedly extracted with diethyl ether. The combined organic phases were dried over MgSO_4_, filtered and concentrated in vacuo. The residue was purified by column chromatography (petroleum ether-Et_2_O 19:1) to afford **5e**–**g** as colourless oils.

*(1-Methylsulfanyl)-8-nitrooctane* **5e**. Colourless oil (3.2 g, 65% yield). ^1^H-NMR (CDCl_3_) δ 1.23–1.42 (m, 8H, H-3-6), 1.57 (qt, 2H, H-2), 1.98 (qt, 2H, H-7), 2.07 (s, 3H, MeS), 2.46 (t, 2H, *J*_vic_ = 7.3, H-1), 4.36 (t, 2H, *J*_vic_ = 7.3, H-8). ^13^C-NMR δ 15.3 (MeS), 25.9 (C-4), 27.1 (C-7), 28.3, 28.5, 28.6, (C-3, C-5, C-6), 28.8 (C-2), 34.0 (C-1), 75.6 (C-8). MS IS *m*/*z* 206.3 [M + H]^+^.

*(1-Methylsulfanyl)-10-nitrodecane* **5f**. Colourless oil (3.8 g, 68% yield). ^1^H-NMR (CDCl_3_) δ 1.21–1.41 (m, 12H, H-3-8), 1.57 (qt, 2H, H-2), 1.99 (qt, 2H, H-9), 2.07 (s, 3H, MeS), 2.47 (t, 2H, *J*_vic_ = 7.3, H-1), 4.36 (t, 2H, *J*_vic_ = 7.3, H-10). ^13^C-NMR δ 15.3 (MeS), 26.0, 27.2, 28.5, 28.6, 28.9, 29.0 (C-3-9), 29.1 (C-2), 34.1 (C-1), 75.6 (C-10). MS IS *m*/*z* 234.4 [M + H]^+^.

*(1-Methylsulfanyl)-12-nitrododecane* **5g**. Colourless oil (4.2 g, 67% yield). ^1^H-NMR (CDCl_3_) δ 1.21–1.42 (m, 16H, H-3-10), 1.58 (qt, 2H, H-2), 2.0 (qt, 2H, H-11), 2.09 (s, 3H, MeS), 2.48 (t, 2H, *J*_vic_ = 7.3, H-1), 4.37 (t, 2H, *J*_vic_ = 7.3, H-12). ^13^C-NMR δ 15.3 (MeS), 26.0, 27.2, 28.6, 28.9, 29.2 (C-3-11), 29.3 (C-2), 34.1 (C-1), 75.6 (C-12). MS IS *m*/*z* 262.4 [M + H]^+^.

#### 3.2.4. General Procedure for the Nitronate Chlorination and Coupling with the Thioglucose Unit

*Nitronate formation*. To a stirred freshly prepared solution of sodium (0.3 g) in 2-butanol (17 mL), under argon atmosphere, a solution of nitro derivative **5** (13 mmol) in anhydrous ether (20 mL) is added dropwise under exclusion of moisture. The slow addition of anhydrous ether (200 mL) while stirring caused precipitation of the nitronate as a white solid. After 10 min more stirring, the suspension was rapidly filtered off on sintered glass, and, after rinsing with anhydrous ether (20–30 mL), the nitronate cake was dried in vacuo for 30 min (preparation of nitronates can be more confortably realized in a glovebox).

*Conversion of nitronate into hydroximoyl chloride*. The powdered nitronate obtained was suspended in dry chloroform (40 mL), the stirred mixture was cooled at −60 °C, and a chloroform solution of freshly distilled thionyl chloride (1 mL in 5 mL) was added dropwise. After 20 min more stirring at −60°C, the reaction was quenched by pouring the mixture into ice water; the chloroform solution was separated and the aqueous phase was extracted twice with chloroform. The combined organic phases were dried over MgSO_4_, filtered and concentrated in vacuo to afford the raw hydroximoyl chloride as a greenish oil.

*Coupling with the thioglucose unit*. This oily residue was immediately dissolved in 70 mL of a dry dichloromethane–diethyl ether mixture (2:1 *v*/*v*), 2,3,4,6-tetra-*O*-acetyl-1-thio-β-d-glucopyranose (3.64 g, 10 mmol) was added, then dry argon was bubbled for 15 min in the solution cooled to −10 °C. A solution of triethylamine (4.2 mL, 30 mmol) in dichloromethane (10 mL) was added in one portion and the mixture was stirred during 45 min more. After washing with ice-cold 1N sulfuric acid, the organic phase was separated and the aqueous phase extracted twice with dichloromethane. The combined organic phases were dried over MgSO_4_, filtered and concentrated in vacuo. The syrupy residue was purified by column chromatography (petroleum ether–EtOAc 3:2) to afford the glycosyl thiohydroximates **18** as amorphous solids.

*S-(2,3,4,6-Tetra-O-acetyl-β-d-glucopyranosyl) (Z)-(3′-methylsulfanyl)propanethiohydroximate* **18a**. White amorphous powder (32% yield), [α]_D_ −20 (*c* = 1.0, CHCl_3_). ^1^H-NMR (CDCl_3_) δ 2.00, 2.03, 2.05, 2.10, (4s, 12H, CH_3_CO), 2.14 (s, 3H, MeS), 2.78–2.81 (m, 4H, H-2′, H-3′), 3.78 (dt, 1H, *J*_4–5_ = 10.0, H-5), 4.17 (d, 2H, *J*_5–6a_ = *J*_5–6b_ = 3.9, H-6a, H-6b), 5.05–5.12 (m, 3H, H-1, H-2, H-4), 5.24–5.30 (m, 1H, H-3), 8.04 (brs, NOH). ^13^C-NMR δ 15.5 (MeS), 20.3, 20.4, 20.6 (CH_3_CO), 31.1 (C-2′), 32.7 (C-1′), 62.0 (C-6), 67.9 (C-4), 70.0 (C-2), 73.6 (C-3), 75.9 (C-5), 79.9 (C-1), 150.8 (C=N), 169.4, 169.5, 170.3, 170.8 (C=O). HR-ESI-MS: C_18_H_27_NO_10_S_2_: calcd. 481.1076; found 481.1061.

*S-(2,3,4,6-Tetra-O-acetyl-β-d-glucopyranosyl) (Z)-(4′-methylsulfanyl)butanethiohydroximate* **18b**. White amorphous powder (34% yield), [α]_D_ −16 (*c* = 1.0, CHCl_3_). ^1^H-NMR (CDCl_3_) δ 1.90–1.95 (m, 2H, H-3′), 1.99, 2.01, 2.03, 2.06 (4s, 12H, *CH_3_*CO), 2.10 (s, 3H, MeS), 2.52–2.67 (m, 4H, H-2′, H-4′), 3.82 (ddd, 1H, *J*_4–5_ = 9.9, H-5), 4.13 (dd, 1H, *J*_5–6b_ = 2.4, *J*_gem_ = 11.8, H-6b), 4.20 (dd, 1H, *J*_5–6a_ = 5.0, H-6a), 5.00–5.12 (m, 3H, H-1, H-2, H-4), 5.24 (t, 1H, *J*_3–4_ = 9.0, H-3), 8.80 (brs, NOH). ^13^C-NMR δ 15.4 (MeS), 20.4, 20.5, 20.6 (CH_3_CO), 26.1 (C-3′), 30.9 (C-4′), 33.4 (C-2′), 62.0 (C-6), 67.9 (C-4), 70.1 (C-2), 73.8 (C-3), 75.8 (C-5), 79.8 (C-1), 151.7 (C=N), 169.3, 169.5, 170.4, 170.8 (C=O). HR-ESI-MS: C_19_H_29_NO_10_S_2_: calcd. 495.1233; found 495.1230.

*S-(2,3,4,6-Tetra-O-acetyl-β-d-glucopyranosyl) (Z)-(5′-methylsulfanyl)pentanethiohydroximate* **18c**. White amorphous powder (40% yield), [α]_D_ −17 (*c* = 1.0, CHCl_3_). ^1^H-NMR (CDCl_3_) δ 1.60–1.85 (m, 4H, H-3′, H-4′), 2.02, 2.05, 2.07, 2.09 (4s, 12H, *CH_3_*CO), 2.11 (s, 3H, MeS), 2.45–2.55 (m, 4H, H-2′, H-5′), 3.79 (ddd, 1H, *J*_4–5_ = 9.7, H-5), 4.14 (dd, 1H, *J*_5–6b_ = 2.5, *J*_gem_ = 12.6, H-6b), 4.22 (dd, 1H, *J*_5–6a_ = 5.4, H-6a), 5.05–5.15 (m, 3H, H-1, H-2, H-4), 5.25 (dd, 1H, *J*_3–4_ = 9.3, H-3), 8.20 (brs, NOH). ^13^C-NMR δ 15.3 (MeS), 20.4, 20.5 (CH_3_CO), 25.8 (C-4′), 28.0 (C-3′), 31.9 (C-5′), 33.5 (C-2′), 62.1 (C-6), 68.0 (C-4), 70.1 (C-2), 73.7 (C-3), 75.8 (C-5), 79.8 (C-1), 152.0 (C=N), 169.3, 169.5, 170.3, 170.8 (C=O). HR-ESI-MS: C_20_H_31_NO_10_S_2_: calcd. 509.1389; found 509.1378.

*S-(2,3,4,6-Tetra-O-acetyl-β-d-glucopyranosyl) (Z)-(6′-methylsulfanyl)hexanethiohydroximate* **18d**. White amorphous powder (48% yield), [α]_D_ −11 (*c* = 1.0, CHCl_3_). ^1^H-NMR (CDCl_3_) δ 1.40–1.51 (m, 2H, H-4′), 1.58–1.72 (m, 4H, H-3′, H-5′), 2.01, 2.02, 2.04, 2.08 (4s, 12H, *CH_3_*CO), 2.10 (s, 3H, MeS), 2.50 (t, 4H, *J*_vic_ = 7.1, H-2′, H-6′), 3.75 (ddd, 1H, *J*_4–5_ = 10.1, H-5), 4.11 (dd, 1H, *J*_5–6b_ = 2.4, *J*_gem_ = 12.3, H-6b), 4.20 (dd, 1H, *J*_5–6a_ = 5.6, H-6a), 5.04–5.12 (m, 3H, H-1, H-2, H-4), 5.27 (dd, 1H, *J*_3–4_ = 9.3, H-3), 8.20 (brs, NOH). ^13^C-NMR δ 15.3 (MeS), 20.4, 20.5 (CH_3_CO), 26.4 (C-4′), 28.0 (C-5′), 28.5 (C-3′), 32.3 (C-6′), 33.8 (C-2′), 62.1 (C-6), 68.0 (C-4), 70.0 (C-2), 73.7 (C-3), 75.9 (C-5), 79.8 (C-1), 152.1 (C=N), 169.3, 169.5, 170.5, 170.7 (C=O). HR-ESI-MS: C_21_H_33_NO_10_S_2_: calcd. 523.1546; found 523.1541.

*S-(2,3,4,6-Tetra-O-acetyl-β-d-glucopyranosyl) (Z)-(8′-methylsulfanyl)octanethiohydroximate* **18e**. White amorphous powder (47% yield), [α]_D_ −11 (*c* = 1.0, CHCl_3_). ^1^H-NMR (CDCl_3_) δ 1.32–1.40 (m, 6H, H-4′, H-5′, H-6′), 1.47–1.64 (m, 4H, H-3′, H-7′), 1.99, 2.01, 2.02, 2.05 (4s, 12H, *CH_3_*CO), 2.07 (s, 3H, MeS), 2.41–2.46 (m, 4H, H-2′, H-8′), 3.73 (ddd, 1H, *J*_4–5_ = 10.0, H-5), 4.09 (dd, 1H, *J*_5–6b_ = 2.1, *J*_gem_ = 12.3, H-6b), 4.18 (dd, 1H, *J*_5–6a_ = 5.4, H-6a), 5.02–5.10 (m, 3H, H-1, H-2, H-4), 5.25 (dd, 1H, *J*_3–4_ = 9.4, H-3), 8.83 (brs, NOH). ^13^C-NMR δ 15.3 (MeS), 20.4, 20.5 (CH_3_CO), 26.8, 28.5, 28.7 (C-4′, C-5′, C-6′), 28.8 (C-7′), 28.9 (C-3′), 32.3 (C-8′), 34.5 (C-2′), 62.1 (C-6), 68.0 (C-4), 70.0 (C-2), 73.7 (C-3), 75.9 (C-5), 79.8 (C-1), 152.2 (C=N), 169.3, 169.5, 170.4, 170.7 (C=O). HR-ESI-MS: C_23_H_37_NO_10_S_2_: calcd. 551.1859; found 551.1851.

*S-(2,3,4,6-Tetra-O-acetyl-β-d-glucopyranosyl) (Z)-(10′-methylsulfanyl)decanethiohydroximate* **18f**. White amorphous powder (51% yield), [α]_D_ −12 (*c* = 1.0, CHCl_3_). ^1^H-NMR (CDCl_3_) δ 1.28–1.34 (m, 10H, H-4′ to H-8′), 1.51–1.63 (m, 4H, H-3′, H-9′), 1.98, 2.01, 2.02, 2.04 (4s, 12H, *CH_3_*CO), 2.06 (s, 3H, MeS), 2.38–2.47 (m, 4H, H-2′, H-10′),3.71 (ddd, 1H, *J*_4–5_ = 10.0, H-5), 4.08 (dd, 1H, *J*_5–6b_ = 2.4, *J*_gem_ = 12.1, H-6b), 4.19 (dd, 1H, *J*_5–6a_ = 5.2, H-6a), 5.00–5.09 (m, 3H, H-1, H-2, H-4), 5.22 (dd, 1H, *J*_3–4_ = 9.2, H-3), 8.86 (brs, NOH). ^13^C-NMR δ 15.3 (MeS), 20.4, 20.5 (CH_3_CO), 26.9, 28.6, 28.9, (C-4′ to C-8′), 29.0 (C-9′), 29.2 (C-3′), 32.4 (C-10′), 34.1 (C-2′), 62.1 (C-6), 68.0 (C-4), 70.0 (C-2), 73.7 (C-3), 75.9 (C-5), 79.8 (C-1), 152.3 (C=N), 169.3, 169.5, 170.4, 170.7 (C=O). HR-ESI-MS: C_25_H_41_NO_10_S_2_: calcd. 579.2172; found 579.2163.

*S-(2,3,4,6-Tetra-O-acetyl-β-d-glucopyranosyl) (Z)-(12′-methylsulfanyl)dodecanethiohydroximate* **18g**. White amorphous powder (56% yield), [α]_D_ −12 (*c* = 1.0, CHCl_3_). ^1^H-NMR (CDCl_3_) δ 1.31–1.38 (m, 14H, H-4′ to H-10′), 1.53–1.66 (m, 4H, H-3′, H-11′), 1.98, 2.01, 2.02, 2.05 (4s, 12H, CH_3_CO), 2.07 (s, 3H, MeS), 2.40–2.45 (m, 4H, H-2′, H-12′), 3.72 (ddd, 1H, *J*_4–5_ = 9.8, H-5), 4.09 (dd, 1H, *J*_5–6b_ = 2.5, *J*_gem_ = 12.5, H-6b), 4.19 (dd, 1H, *J*_5–6a_ = 5.5, H-6a), 5.01–5.12 (m, 3H, H-1, H-2, H-4), 5.24 (dd, 1H, *J*_3–4_ = 9.5, H-3), 8.88 (brs, NOH). ^13^C-NMR δ 15.3 (MeS), 20.4, 20.5 (CH_3_CO), 26.7, 28.9, 29.0 (C-4′ to C-10′), 29.1 (C-11′), 29.2 (C-3′), 32.4 (C-12′), 34.3 (C-2′), 62.1 (C-6), 67.9 (C-4), 70.0 (C-2), 73.6 (C-3), 75.8 (C-5), 79.8 (C-1), 152.3 (C=N), 169.2, 169.5, 170.3, 170.6 (C=O). HR-ESI-MS: C_27_H_45_NO_10_S_2_: calcd. 607.2485; found 607.2478.

#### 3.2.5. General Procedure for *NO*-Sulfation of the Glucosyl Thiohydroximates **18**

Sulfur trioxide pyridine complex (400 mg, 5 eq.) was added to a solution of compound **18** (0.5 mmol) in dimethylformamide (7 mL). After 24 h stirring at rt, the reaction mixture was treated with a 0.2 M aqueous solution of KHCO_3_ (12 mL) then the solvents were evaporated in vacuo. Chromatographic purification (CH_2_Cl_2_/MeOH 17:3) provided compounds **19**.

*Per-O-acetylated 2-methylsulfanylethyl glucosinolate* **19a**. White amorphous powder (61% yield), [α]_D_ −20 (*c* = 0.9, MeOH). ^1^H-NMR (DMSO-*d*_6_) δ 1.96, 1.99, 2.01, 2.03 (4s, 12H, CH_3_CO), 2.23 (s, 3H, MeS), 2.46–2.53 (m, 4H, H-1′, H-2′), 4.03–4.15 (m, 3H, H-5, H-6a, H-6b), 4.88 (t, 1H, *J*_2–3_ = 9.4, H-2), 4.94 (t, 1H, *J*_4–5_ = 8.6, H-4), 5.39 (dd, 1H, *J*_3–4_ = 9.6, H-3), 5.56 (d, 1H, *J*_1–2_ = 10.1, H-1). ^13^C-NMR δ 14.6 (MeS), 20.2, 20.3, 20.5 (CH_3_CO), 26.5 (C-2′), 32.9 (C-1′), 62.2 (C-6), 68.1 (C-4), 69.8 (C-2), 72.9 (C-3), 74.0 (C-5), 78.3 (C-1), 153.2 (C=N), 169.4, 169.7, 170.0, 170.3 (C=O). HR-ESI-MS: C_18_H_26_NO_13_S_3_: calcd. 560.0566; found 560.0571.

*Per-O-acetylated 3-methylsulfanylpropyl glucosinolate* **19b**. White amorphous powder (68% yield), [α]_D_ −22 (*c* = 1.1, MeOH). ^1^H-NMR (DMSO-*d*_6_) δ 1.79–1.90 (m, 2H, H-2′), 1.94, 1.97, 1.99, 2.00 (4s, 12H, CH_3_CO), 2.05 (s, 3H, MeS), 2.53 (t, 2H, *J*_vic_ = 7.3, H-3′), 2.63 (t, 2H, *J*_vic_ = 7.3, H-1′), 4.00–4.18 (m, 3H, H-5, H-6a, H-6b), 4.86 (t, 1H, *J*_2–3_ = 9.0, H-2), 4.89 (t, 1H, *J*_4–5_ = 8.8, H-4), 5.43 (dd, 1H, *J*_3–4_ = 9.3, H-3), 5.50 (d, 1H, *J*_1–2_ = 10.2, H-1). ^13^C-NMR δ 14.7 (MeS), 20.2, 20.3, 20.5 (CH_3_CO), 26.4 (C-3′), 30.3, 32.5 (C-1′, C-2′), 62.1 (C-6), 68.0 (C-4), 69.6 (C-2), 72.8 (C-3), 74.4 (C-5), 78.2 (C-1), 153.5 (C=N), 169.4, 169.6, 169.9, 170.3 (C=O). HR-ESI-MS: C_19_H_28_NO_13_S_3_: calcd. 574.0723; found 574.0730.

*Per-O-acetylated 4-methylsulfanylbutyl glucosinolate* **19c**. White amorphous powder (86% yield), [α]_D_ −18 (*c* = 1.0, MeOH). ^1^H-NMR (DMSO-*d*_6_) δ 1.52–1.68 (m, 4H, H-2′, H-3′), 1.94, 1.97, 1.99, 2.00 (4s, 12H, CH_3_CO), 2.02 (s, 3H, MeS), 2.48–2.59 (m, 4H, H-1′, H-4′), 4.00–4.15 (m, 3H, H-5, H-6a, H-6b), 4.86 (t, 1H, *J*_2–3_ = 9.5, H-2), 4.91 (t, 1H, *J*_4–5_ = 8.5, H-4), 5.43 (dd, 1H, *J*_3–4_ = 9.5, H-3), 5.50 (d, 1H, *J*_1–2_ = 10.2, H-1). ^13^C-NMR δ 14.5 (MeS), 20.2, 20.3, 20.5 (CH_3_CO), 25.7 (C-4′), 27.9, 31.0, 32.8 (C-1′, C-2′, C-3′), 62.2 (C-6), 68.1 (C-4), 69.6 (C-2), 72.8 (C-3), 74.4 (C-5), 78.2 (C-1), 154.0 (C=N), 169.4, 169.6, 169.9, 170.3 (C=O). HR-ESI-MS: C_20_H_30_NO_13_S_3_: calcd. 588.0879; found 588.0887.

*Per-O-acetylated 5-methylsulfanylpentyl glucosinolate* **19d**. White amorphous powder (84% yield), [α]_D_ −15 (*c* = 1.1, MeOH). ^1^H-NMR (DMSO-*d*_6_) δ 1.38–1.68 (m, 6H, H-2′, H-3′, H-4′), 1.94, 1.98, 1.99, 2.00 (4s, 12H, CH_3_CO), 2.02 (s, 3H, MeS), 2.43–2.57 (m, 4H, H-1′, H-5′), 3.98–4.15 (m, 3H, H-5, H-6a, H-6b), 4.86 (t, 1H, *J*_2–3_ = 9.8, H-2), 4.91 (t, 1H, *J*_4–5_ = 8.8, H-4), 5.45 (dd, 1H, *J*_3–4_ = 9.7, H-3), 5.51 (d, 1H, *J*_1–2_ = 10.2, H-1). ^13^C-NMR δ 15.6 (MeS), 20.2, 20.4, 20.5 (CH_3_CO), 26.4 (C-5′), 27.8, 28.2, 31.4, 33.2 (C-1′ to C-4′), 62.2 (C-6), 68.2 (C-4), 69.6 (C-2), 72.8 (C-3), 74.4 (C-5), 78.2 (C-1), 154.1 (C=N), 169.4, 169.6, 169.9, 170.3 (C=O). HR-ESI-MS: C_21_H_32_NO_13_S_3_: calcd. 602.1036; found 602.1041.

*Per-O-acetylated 7-methylsulfanylheptyl glucosinolate* **19e**. White amorphous powder (83% yield), [α]_D_ −17 (*c* = 1.0, MeOH). ^1^H-NMR (DMSO-*d*_6_) δ 1.24–1.39 (m, 6H, H-3′, H-4′, H-5′), 1.44–1.65 (m, 4H, H-2′, H-6′), 1.94, 1.97, 1.99, 2.01 (4s, 12H, CH_3_CO), 2.01 (s, 3H, MeS), 2.40–2.54 (m, 4H, H-1′, H-7′), 3.98–4.15 (m, 3H, H-5, H-6a, H-6b), 4.85 (t, 1H, *J*_2–3_ = 9.2, H-2), 4.91 (t, 1H, *J*_4–5_ = 8.8, H-4), 5.46 (dd, 1H, *J*_3–4_ = 9.4, H-3), 5.49 (d, 1H, *J*_1–2_ = 10.3, H-1). ^13^C-NMR δ 14.7 (MeS), 20.2, 20.3, 20.4 (CH_3_CO), 26.8 (C-7′), 28.2, 28.3, 28.6, 31.4, 33.2 (C-1′ to C-6′), 62.2 (C-6), 68.2 (C-4), 69.6 (C-2), 72.8 (C-3), 74.4 (C-5), 78.2 (C-1), 154.1 (C=N), 169.4, 169.6, 169.9, 170.2 (C=O). HR-ESI-MS: C_23_H_36_NO_13_S_3_: calcd. 630.1049; found 630.1060.

*Per-O-acetylated 9-methylsulfanylnonyl glucosinolate* **19f**. White amorphous powder (85% yield), [α]_D_ −16 (*c* = 1.0, MeOH). ^1^H-NMR (DMSO-*d*_6_) δ 1.21–1.40 (m, 10H, H-3′ to H-7′), 1.44–1.59 (m, 4H, H-2′, H-8′), 1.95, 1.97, 1.98, 2.00 (4s, 12H, CH_3_CO), 2.01 (s, 3H, MeS), 2.40–2.54 (m, 4H, H-1′, H-9′), 3.98–4.14 (m, 3H, H-5, H-6a, H-6b), 4.84 (t, 1H, *J*_2–3_ = 9.7, H-2), 4.90 (t, 1H, *J*_4–5_ = 8.5, H-4), 5.46 (dd, 1H, *J*_3–4_ = 9.7, H-3), 5.50 (d, 1H, *J*_1–2_ = 10.0, H-1). ^13^C-NMR δ 15.1 (MeS), 20.2, 20.3, 20.4 (CH_3_CO), 26.9 (C-9′), 28.2, 28.4, 28.5, 28.8, 29.1, 31.6, 34.3 (C-1′ to C-8′), 62.2 (C-6), 68.4 (C-4), 70.0 (C-2), 73.1 (C-3), 74.7 (C-5), 78.7 (C-1), 154.5 (C=N), 169.3, 169.5, 170.0, 170.3 (C=O). HR-ESI-MS: C_25_H_40_NO_13_S_3_: calcd. 658.1662; found 658.1665.

*Per-O-acetylated 11-methylsulfanylundecyl glucosinolate* **19g**. White amorphous powder (84% yield), [α]_D_ −16 (*c* = 1.0, MeOH). ^1^H-NMR (DMSO-*d*_6_) δ 1.24–1.40 (m, 14H, H-3′ to H-9′), 1.42–1.63 (m, 4H, H-2′, H-10′), 1.96, 1.98, 2.00, 2.02 (4s, 12H, CH_3_CO), 2.01 (s, 3H, MeS), 2.47–2.56 (m, 4H, H-1′, H-11′), 3.93–4.13 (m, 3H, H-5, H-6a, H-6b), 4.86 (t, 1H, *J*_2–3_ = 9.6, H-2), 4.93 (t, 1H, *J*_4–5_ = 8.8, H-4), 5.47 (dd, 1H, *J*_3–4_ = 9.5, H-3), 5.51 (d, 1H, *J*_1–2_ = 10.0, H-1). ^13^C-NMR δ 14.71 (MeS), 20.2, 20.3, 20.4 (CH_3_CO), 26.5 (C-11′), 28.8, 29.0, 29.3, 29.5, 29.9, 31.5, 32.4, 34.8 (C-1′ to C-10′), 62.1 (C-6), 68.4 (C-4), 69.8 (C-2), 73.2 (C-3), 75.1 (C-5), 78.3 (C-1), 154.5 (C=N), 169.4, 169.6, 170.0, 170.3 (C=O). HR-ESI-MS: C_27_H_44_NO_13_S_3_: calcd. 686.1975; found 686.1987.

#### 3.2.6. General Procedure for Deprotection of the Glucopyranosyl Moiety

To a suspension of peracetylated glucosinolate **19** (0.1 mmol) in dry methanol (5 mL) under argon, a freshly prepared 1 M solution of potassium methoxide was added dropwise until the pH reached 9. After 4 h standing at rt, the reaction mixture was neutralized by the addition of Dowex H^+^ resin. After filtration and evaporation in vacuo, the resulting crude was purified by C-18 silica gel column chromatography (eluent: water) and freeze-drying to provide glucosinolates **2**.

*2-Methylsulfanylethyl glucosinolate [27303-30-6]* **2a**. White amorphous powder (80% yield), [α]_D_ −27 (*c* = 0.95, H_2_O). ^1^H-NMR (D_2_O) δ 2.19 (s, 3H, MeS), 2.92–2.98 (m, 2H, H-2′), 3.04–3.10 (m, 2H, H-1′), 3.44–3.53 (m, 2H, H-2, H-4), 3.58–3.69 (m, 2H, H-3, H-5), 3.76 (dd, 1H, *J*_5–6b_ = 5.8, *J*_gem_ = 13.0, H-6b), 3.95 (dd, 1H, *J*_5–6a_ = 2.6, H-6a), 5.09 (d, 1H, *J*_1–2_ = 10.1, H-1). ^13^C-NMR δ 15.3 (MeS), 30.5 (C-2′), 33.0 (C-1′), 61.5 (C-6), 70.2 (C-4), 73.5 (C-2), 78.3 (C-3), 81.5 (C-5), 83.1 (C-1), 164.9 (C=N). HR-ESI-MS: C_10_H_18_NO_9_S_3_: calcd. 392.0144; found 392.0133.

*3-Methylsulfanylpropyl glucosinolate [26888-03-9]* **2b**. **Glucoibervirin**. White amorphous powder (84% yield), [α]_D_ −21 (*c* = 1.05, H_2_O). ^1^H-NMR (D_2_O) δ 2.05 (qt, 2H, H-2′), 2.16 (s, 3H, MeS), 2.67 (t, 2H, *J*_vic_ = 7.5, H-3′), 2.63 (t, 2H, *J*_vic_ = 7.5, H-1′), 3.46–3.52 (m, 2H, H-2, H-4), 3.55–3.66 (m, 2H, H-3, H-5), 3.73 (dd, 1H, *J*_5–6b_ = 5.8, *J*_gem_ = 13.2, H-6b), 3.93 (dd, 1H, *J*_5–6a_ = 2.7, H-6a), 5.09 (d, 1H, *J*_1–2_ = 10.1, H-1). ^13^C-NMR δ 15.3 (MeS), 26.7 (C-2′), 30.9 (C-3′), 34.1 (C-1′), 61.4 (C-6), 70.7 (C-4), 73.2 (C-2), 78.2 (C-3), 82.5 (C-5), 83.8 (C-1), 165.1 (C=N). HR-ESI-MS: C_11_H_20_NO_9_S_3_: calcd. 406.0300; found 406.0291.

*4-Methylsulfanylbutyl glucosinolate [21973-56-8]* **2c**. **Glucoerucin**. White amorphous powder (90% yield), [α]_D_ −20 (*c* = 1.00, H_2_O). ^1^H-NMR (D_2_O) δ 1.67–1.89 (m, 4H, CH_2_), 2.12 (s, 3H, MeS), 2.61 (t, 2H, *J*_vic_ = 7.5, H-4′), 2.76 (t, 2H, *J*_vic_ = 7.5, H-1′), 3.42–3.51 (m, 2H, H-2, H-4), 3.57–3.63 (m, 2H, H-3, H-5), 3.72 (dd, 1H, *J*_5–6b_ = 5.8, *J*_gem_ = 13.1, H-6b), 3.91 (dd, 1H, *J*_5–6a_ = 2.7, H-6a), 5.06 (d, 1H, *J*_1–2_ = 10.1, H-1). ^13^C-NMR δ 15.2 (MeS), 25.7 (C-2′), 28.2 (C-3′), 31.6 (C-4′), 33.0 (C-1′), 62.2 (C-6), 71.2 (C-4), 73.5 (C-2), 78.7 (C-3), 81.8 (C-5), 83.0 (C-1), 165.4 (C=N) [47]. HR-ESI-MS: C_12_H_22_NO_9_S_3_: calcd. 420.0457; found 420.0453.

*5-Methylsulfanylpentyl glucosinolate [29611-01-6]* **2d**. **Glucoberteroin**. White amorphous powder (90% yield), [α]_D_ −21 (*c* = 0.95, H_2_O). ^1^H-NMR (D_2_O) δ 1.44 (qt, 2H, *J*_vic_ = 7.4, H-3′), 1.61 (qt, 2H, *J*_vic_ = 7.3, H-4′) 1.70 (qt, 2H, *J*_vic_ = 7.3, H-2′), 2.06 (s, 3H, MeS), 2.53 (t, 2H, *J*_vic_ = 7.3, H-5′), 2.68 (t, 2H, *J*_vic_ = 7.3, H-1′), 3.38–3.45 (m, 2H, H-2, H-4), 3.50–3.56 (m, 2H, H-3, H-5), 3.68 (dd, 1H, *J*_5–6b_ = 5.8, *J*_gem_ = 13.1, H-6b), 3.85 (dd, 1H, *J*_5–6a_ = 2.6, H-6a), 5.00 (d, 1H, *J*_1–2_ = 10.0, H-1). ^13^C-NMR δ 15.1 (*Me*S), 27.5 (C-3′), 28.5 (C-2′), 29.0 (C-4′), 33.0 (C-5′), 34.2 (C-1′), 61.8 (C-6), 70.3 (C-4), 73.3 (C-2), 78.4 (C-3), 81.4 (C-5), 83.0 (C-1), 164.5 (C=N) [48].HR-ESI-MS: C_13_H_24_NO_9_S_3_: calcd. 434.0613; found 434.0610.

*7-Methylsulfanylheptyl glucosinolate [80667-67-0]* **2e**. White amorphous powder (90% yield), [α]_D_ −22 (*c* = 1.00, H_2_O). ^1^H-NMR (D_2_O) δ 1.24–1.39 (m, 6H, CH_2_), 1.44–1.65 (m, 4H, CH_2_), 2.01 (s, 3H, MeS), 2.38–2.54 (m, 4H, CH_2_), 3.45–3.52 (m, 2H, H-2, H-4), 3.55–3.63 (m, 2H, H-3, H-5), 3.68 (dd, 1H, *J*_5–6b_ = 5.7, *J*_gem_ = 12.8, H-6b), 3.89 (dd, 1H, *J*_5–6a_ = 2.6, H-6a), 5.06 (d, 1H, *J*_1–2_ = 10.0, H-1). ^13^C-NMR δ 15.1(MeS), 27.8, 28.7, 28.9 (C-3′-C-5′), 29.0 (C-2′), 29.2 (C-6′), 33.0 (C-1′), 34.3 (C-7′), 61.6 (C-6), 70.1 (C-4), 73.0 (C-2), 78.2 (C-3), 81.2 (C-5), 82.9 (C-1), 165.4 (C=N). HR-ESI^-^-MS: C_15_H_28_NO_9_S_3_: calcd. 462.0926; found 462.0932 [48]. HR-ESI-MS: C_15_H_28_NO_9_S_3_: calcd. 462.0926; found 462.0919.

*9-Methylsulfanylnonyl glucosinolate [81149-01-1]* **2f**. White amorphous powder (93% yield), [α]_D_ −19 (*c* = 0.84, H_2_O). ^1^H-NMR (D_2_O) δ 1.21–1.40 (m, 10H, CH_2_), 1.44–1.59 (m, 4H, CH_2_), 2.00 (s, 3H, MeS), 2.43–2.58 (m, 4H, (CH_2_), 3.40–3.51 (m, 2H, H-2, H-4), 3.57–3.62 (m, 2H, H-3, H-5), 3.71 (dd, 1H, *J*_5–6b_ = 5.7, *J*_gem_ = 13.0, H-6b), 3.91 (dd, 1H, *J*_5–6a_ = 2.7, H-6a), 5.06 (d, 1H, *J*_1–2_ = 10.1, H-1). ^13^C-NMR δ 15.4 (MeS), 26.9, 28.2, 28.4, 28.6 (C-3′-C-7′), 28.9 (C-2′), 29.2 (C-8′), 31.9 (C-1′), 34.6 (C-9′), 61.4 (C-6), 70.5 (C-4), 73.4 (C-2), 78.3 (C-3), 82.2 (C-5), 83.5 (C-1), 164.8 (C=N). HR-ESI-MS: C_17_H_32_NO_9_S_3_: calcd. 490.1239; found 490.1231.

*11-Methylsulfanylundecyl glucosinolate* **2g**. Colourless gum (91% yield), [α]_D_ −21 (*c* = 0.92, H_2_O). ^1^H-NMR δ (D_2_O) 1.26–1.44 (m, 10H, CH_2_), 1.42–1.63 (m, 4H, (CH_2_), 2.02 (s, 3H, MeS), 2.44–2.56 (m, 4H, (CH_2_), 3.39–3.50 (m, 2H, H-2, H-4), 3.54–3.61 (m, 2H, H-3, H-5), 3.74 (dd, 1H, *J*_5–6b_ = 5.8, *J*_gem_ = 13.0, H-6b), 3.93 (dd, 1H, *J*_5–6a_ = 2.6,, H-6a), 5.06 (d, 1H, , *J*_1–2_ = 10.1, H-1). ^13^C-NMR δ 15.2 (MeS), 26.5, 28.9, 29.0, 29.5 (C3′-C-9′), 29.7 (C-2′), 30.0 (C-10′), 32.2 (C-1′), 34.8 (C-11′), 61.6 (C-6), 70.2 (C-4), 73.1 (C-2), 78.7 (C-3), 81.3 (C-5), 83.0 (C-1), 165.4 (C=N). HR-ESI-MS: C_19_H_36_NO_9_S_3_: calcd. 518.1552; found 518.1547.

## 4. Conclusions

We have developed a general pathway for the synthesis of biologically critical ω-methylsulfanylalkyl GLs involving tailor-made ω-methylsulfanyl nitroalkanes precursors. Synthetic conditions to access those key-intermediates were adjusted according to the length of the alkyl chain that is required. Extension of this work to the synthesis of ω-methylsulfinyl and ω-methylsulfonyl GLs is under current development in our research group.

## Figures and Tables

**Table 1:** Preparation of small-sized intermediates.

Chain Size n	cpd n°	Yield (%)	cpd n°	Yield (%)	cpd n°	Yield (%)
3	**7a**	47	**8a** [26]	68	**5a**	60
4	**7b**	72	**8b**	--	**5b**	--
5	**7c**	86	**8c**	--	**5c**	--
6	**7d**	94	**8d**	56	**5d**	74

**Table 2:** Preparation of small-sized chloro-nitro derivatives **11a**–**d**.

Chain Size n	cpd n°	Reaction Time (h)	Nitro/Nitrite Ratio (%)	Isolated Yield (%)
3	**11a**	2.5	46/--	39–42
4	**11b**	4.0	70/15	57–60
5	**11c**	4.5	74/20	64–69
6	**11d**	4.5	76/21	73–78

**Table 3:** Alternative preparation (method B) of small-sized precursors **5**.

Chain Size n	cpd n°	T (°C)	Reaction Time (h)	Yield (%)
3	**5a**	25	40	25
4	**5b**	reflux	1	40
5	**5c**	reflux	3	87
6	**5d**	reflux	4	93
